# Intraoperative Electromyographic Evaluation of Brachial Plexus Decompression During the Roos Surgical Procedure for Thoracic Outlet Syndrome

**DOI:** 10.3390/medicina62020332

**Published:** 2026-02-06

**Authors:** Thrasyvoulos Michos, Anastasia Roumpaki, Emmanouil I. Kapetanakis, Petros Michos, Ioannis Gakidis, Christos Chantziantoniou, Aikaterini Kotroni, Ioanna Vlachou, Asterios Kanakis, Vicenzo Castilletti, Dimitris Lazos, Chara Tzavara, George Babis, Periklis I. Tomos, Spiros Pneumaticos

**Affiliations:** 1 Department of Thoracic Surgery, “Attikon” University Hospital, National and Kapodistrian University of Athens, 124 61 Athens, Greece; periklistomos@hotmail.com; 2 Liver Outpatient Unit, 3rd Internal Medicine Department, Evangelismos General Hospital, 106 76 Athens, Greece; ana_roumpaki@yahoo.com; 3 Department of General Thoracic Surgery, General Hospital of Attica “Κ.A.Τ”, 145 61 Athens, Greece; pmichos@yahoo.gr (P.M.); gakidis1958@gmail.com (I.G.); chatziantoniouc@gmail.com (C.C.); 4 Department of Physical Rehabilitation Medicine, General Hospital of Attica “Κ.A.Τ”, 145 61 Athens, Greece; katekot@otenet.gr (A.K.); asterioskanakis1@gmail.com (A.K.); 5 Radiology Department, General Hospital of Attica “Κ.A.Τ”, 145 61 Athens, Greece; ioannaareti@yahoo.gr; 6 Department of General Thoracic Surgery, 401 Military Hospital of Athens, 115 25 Athens, Greece; castilletiv@yahoo.gr; 7 Getremed Ltd., 116 36 Athens, Greece; dimitris.lazos@getremed.gr; 8 Center of Health Services Research, Department of Hygiene, Epidemiology and Medical Statistics Medical School, National and Kapodistrian University of Athens, 115 27 Athens, Greece; htzavara@med.uoa.gr; 9 2nd Department of Orthopaedics, Konstantopouleio Hospital, National and Kapodistrian University of Athens, 105 59 Athens, Greece; george.babis@gmail.com; 10 3rd Department of Orthopaedics, General Hospital of Attica “Κ.A.Τ”, National and Kapodistrian University of Athens, 145 61 Athens, Greece; spirospneumaticos@gmail.com

**Keywords:** Thoracic Outlet Syndrome, transaxillary first rib resection, intraoperative monitoring and assessment of brachial plexus decompression

## Abstract

*Background and Objectives:* The aim of this retrospective study was to assess brachial plexus decompression throughout the sequential stages of the Roos procedure and to elucidate the role of first rib resection in the surgical management of Thoracic Outlet Syndrome (T.O.S). *Materials and Methods*: A total of 34 patients with a mean age of 34.6 years were included in this retrospective analysis. All patients underwent transaxillary first rib resection following anterior scalenotomy, consistent with the Roos procedure. Intraoperative brachial plexus functionality was assessed using recording electrodes for sensory and motor stimulation on the deltoid, biceps, triceps brachii, and abductor digiti minimi muscles. Mixed linear models with log-transformed data were used to assess changes in muscle measurements across surgical stages, with statistical significance at p less than 0.05. *Results*: The electromyographic values of the deltoid, biceps brachii, triceps brachii, and abductor digiti minimi muscles were significantly higher in the final post-operative neutral position compared to both the post-anterior scalenotomy and initial preoperative neutral positions. No significant differences were observed between the initial preoperative neutral position and the post-anterior scalenotomy values for these muscles. However, the abductor digiti minimi muscle exhibited a trend toward decreased values following anterior scalenotomy in comparison to the initial neutral position. *Conclusions*: Intraoperative outcomes of brachial plexus decompression during the Roos procedure demonstrate that first rib resection contributes to complete decompression of the relevant anatomical structures in Thoracic Outlet Syndrome.

## 1. Introduction

The thoracic outlet is the anatomical region extending laterally from the lateral margin of the first rib to the upper medial mediastinum, and superiorly to the level of the fifth cervical nerve root. This space contains numerous critical anatomical structures, including the scalene muscles, the brachial plexus, and several nerves such as the phrenic, long thoracic, suprascapular, and dorsal scapular nerves. Additionally, the region encompasses the stellate ganglion, the subclavian artery and vein, scalene lymph nodes and the apex of the lung. For surgical purposes, it could be divided into an upper part and a lower one [1,2]. The brachial plexus is a complex network of nerves originating from the anterior rami of the C5 to T1 spinal nerves. It consists of five structural segments: roots, trunks, divisions, cords, and branches [3]. The superior trunk, which is formed by the union of the C5 and C6 nerve roots, contributes to the innervation of shoulder musculature-particularly the deltoid and rotator cuff muscles (via C5) [4]. Additionally, it supports elbow flexion through the biceps brachii and facilitates wrist extension (via C6) [4]. The middle trunk, composed of fibers from the C7 nerve root, primarily contributes to the innervation of the triceps brachii (responsible for elbow extension), as well as muscles involved in wrist flexion and finger extension [4]. The inferior trunk, formed by the convergence of the C8 and T1 nerve roots, is primarily responsible for the motor control of the hand and finger movements [3]. It innervates the abductor digiti minimi muscle of the hand through its terminal branch, the ulnar nerve [4].

A longstanding controversy in the surgical management of Thoracic Outlet Syndrome (T.O.S) is regarding the necessity of first rib resection. Contemporary evidence, however, indicates that the current debate pertains primarily to the choice of surgical approach rather than to the performance of rib resection itself. This is supported by substantial data demonstrating that first rib resection achieves complete decompression of the neurovascular structures, while being associated with lower recurrence and reoperation rates [5,6,7,8]. Moreover, first rib resection has been established as a safe and effective intervention across all subtypes of T.O.S, regardless of underlying etiology [5,6,7]. The validity of this practice is further corroborated by multiple studies assessing the long-term postoperative course of affected patients, which consistently report sustained symptom-free daily function without the necessity for additional interventions to address recurrence [7,9].

The purpose of this study is to evaluate the degree of intraoperative decompression of the brachial plexus at various stages of transaxillary first rib resection following anterior scalenotomy (Roos procedure) in the surgical treatment of T.O.S. The procedures were performed at the Department of General Thoracic Surgery of the General Hospital of Attica “K.A.T”, Athens, Greece by thoracic surgeons specializing in the Roos procedure.

## 2. Materials and Methods

### 2.1. Ethics Statement

The study was approved by the Scientific Review Board of the General Hospital of Attika “K.A.T”, (study approval No. 48939, 30 October 2020). Additionally, a corresponding application was submitted and approved by the Bioethics and Ethics Committee of the Medical School of the National and Kapodistrian University of Athens. All participants in the study provided written informed consent prior to the commencement of the study.

### 2.2. Study Design, Inclusion and Exclusion Criteria

This was a retrospective study involving patients who underwent first rib resection following anterior scalenotomy via a transaxillary approach, in the context of Thoracic Outlet Syndrome. The data collection period encompassed cases included in the study over the last five years, from October 2020 through June 2025.

**Figure 1:**
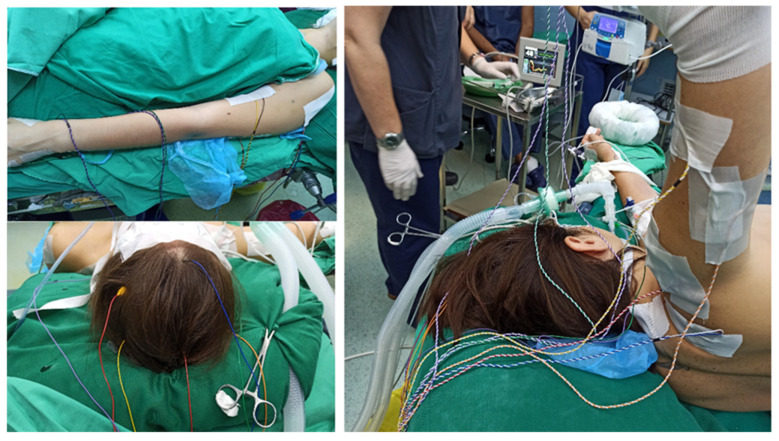
Sites of electrode placement for recording.

Intraoperative evaluation at each stage of the Roos procedure permits a precise assessment of the contribution of first rib resection to overall brachial plexus decompression. The intraoperative assessment was conducted using recording electrodes for sensory and motor stimulation (NIM-ECLIPSE NS ver. 3.5.352, Medtronic, Minneapolis, MN, USA), which were placed on the deltoid, triceps, biceps brachii, and abductor digiti minimi muscles (recording sites 1 and 2, hypothenar eminence and 5th digit of the upper extremity respectively) (Figure 1). Electrodes were placed on the patients’ skull to enable cortical stimulation for the purposes of intraoperative electromyography and motor cortex mapping, with the goal of eliciting muscle responses.

**Figure 2:**
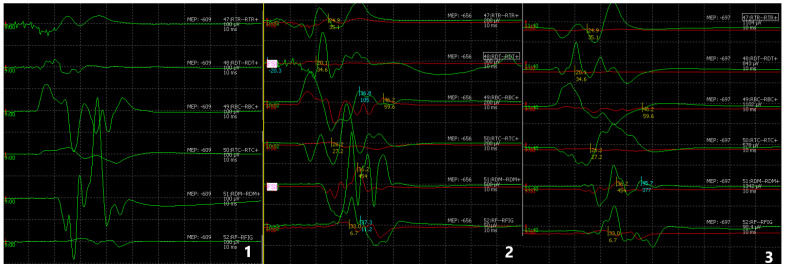
1. Resting preoperative neutral position, 2. After anterior scalenotomy at 90°, 3. Resting post-operative neutral position.

Recordings were initially performed with the limb in the resting position. Subsequently, qualitative and quantitative evaluation of brachial plexus decompression was conducted following anterior scalenotomy, with the limb abducted at 90°, a position required for the transaxillary surgical approach and maintained throughout the procedure. Final measurements were obtained with the limb returned to the resting position after resection of the first rib, marking completion of the surgical intervention. Quantitative assessment was based on the measured difference in current intensity at the aforementioned stages of the operation, as recorded by the electrodes from the respective muscles (Figure 2). In each patient, transcranial cortical stimulation was applied at three predefined stages of the procedure under resting conditions, using a stimulus intensity of 450 V. Motor evoked potentials (MEPs) were recorded at each stage, and changes in MEP amplitude were analyzed as an indirect marker of neural conduction across the brachial plexus. The stimulation was delivered via the cerebral cortex and did not involve direct stimulation of the brachial plexus. The response from each corresponding muscle was recorded and evaluated in μV, the standard unit of measurement of the recording device. No muscle relaxants were administered during intubation and the surgical procedure in order to ensure the accurate recording of neurophysiological potentials. All patients were monitored using the same predefined and standardized neuromonitoring protocol.

The study included patients with T.O.S who underwent surgical treatment via the transaxillary approach. In addition, patients who were previously treated elsewhere using a different surgical approach and subsequently required reoperation due to symptom recurrence and which were managed by the Thoracic Surgery Department of “K.A.T” Hospital as a referral center, were also included. In the aforementioned patients, although a division of the anterior scalene muscle had been previously documented, it had not been performed in practice, given that the anterior scalene muscle was observed to remain intact intraoperatively during the second surgical intervention. Therefore, these patients were included in the study. Patients diagnosed with Double Crush Phenomenon (DCP), in whom the predominant clinical symptomatology was attributed to T.O.S were also included in the study. In these instances, when cervical magnetic resonance imaging demonstrated intervertebral disk herniation, a preoperative assessment by a neurosurgical team was undertaken to ascertain that the presenting symptomatology was not attributable to cervical spine pathology. Furthermore, an orthopedic evaluation was performed to exclude the presence of ulnar neuropathy or carpal tunnel syndrome. Upon completion of these assessments, and in the context of definitive clinical manifestations consistent with T.O.S, patients were deemed appropriate candidates for surgical intervention. Although the recordings from these patients were included in the study, they were excluded from the statistical analyses of the remaining cohort so as to avoid any influence on the final conclusions. Furthermore, the study cohort did not include any patient exhibiting a Pectoralis Minor Syndrome (PMS). Conversely, patients in whom a complete scalenotomy had already been performed elsewhere were excluded from the analysis. Finally, patients who were treated via a supraclavicular approach at the Thoracic Surgery Department of “K.A.T” Hospital were also excluded from the study.

### 2.3. Statistical Analysis

Quantitative variables were expressed as mean values (+/−Standard Deviation) and as median (interquartile range), while categorical variables were expressed as absolute and relative frequencies. In order to evaluate the changes in patients’ muscle measurements throughout the surgery, mixed linear models were performed, taking into account all available measurements regardless missing ones. Regression coefficients (β) with standard errors (SE) were computed from the results of the mixed linear models. Log transformations were used for all muscle measurements due to lack of a normal distribution. All reported *p*-values were two-tailed. Statistical significance was set at *p* < 0.05 and analyses were conducted using STATA statistical software (version 15.0).

## 3. Results

The study’s sample population consisted of 34 patients (82.4% females) with a mean age of 34.6 years (SD = 10.7 years). Their demographic characteristics and clinical presentation are reported in Table 1 and Table 2. Most of the patients (64.7%) reported at most 5 years of symptoms (ranging from 3 months to 20 years, with a mean duration of 5.1 years). Specifically, the mean duration until onset was 3.3 years for the vascular type, 7 years for the neurogenic type, and 5 years for the combined type of T.O.S, respectively. Also, 47.1% had undergone physiotherapy before surgery and 41.2% were physically active. All patients who underwent preoperative physiotherapy reported a transient alleviation of their symptoms for a period of approximately three months; however, due to the subsequent recurrence of symptoms, they ultimately sought further surgical intervention. Multiple compression syndrome (Double Crush Phenomenon—DCP) was present in 17.6% of the cases.

Patients’ muscle measurements for each time point of the surgery separately are presented in Table 3. In order to evaluate the changes in patients’ muscle measurements throughout the surgery, mixed linear models were applied, the results of which are presented in Table 4. Deltoid muscle values were 3.72 times and 4.06 times greater at final post-operative neutral position compared to after anterior scalenotomy (exponentiated β = 3.72, *p* < 0.001) and to initial preoperative neutral position (β = 4.06, *p* < 0.001) respectively. Similarly, biceps brachii muscle values were 2.79-fold and 3.48-fold greater at final post-operative neutral position compared to after anterior scalenotomy (exponentiated β = 2.79, *p* < 0.001) as well as compared to initial preoperative neutral position (exponentiated β = 3.48, *p* = 0.001) respectively. Triceps brachii muscle values were 4.04 and 2.85 times greater at final post-operative neutral position compared to after anterior scalenotomy (exponentiated β = 4.04, *p* < 0.001) as well as compared to initial preoperative neutral position (exponentiated β = 2.85, *p* < 0.001) respectively. Also, abductor digiti minimi muscle 1 and 2 values were 4.70 and 2.07 times greater, respectively, at final post-operative neutral position compared to after anterior scalenotomy (exponentiated β = 4.70, *p* < 0.001 and β = 2.07, *p* = 0.007 respectively) and 1.64 and 1.85 times greater, respectively, at final post-operative neutral position compared to initial preoperative neutral position (exponentiated β = 1.64, *p* = 0.048 and β = 1.85, *p* = 0.020 respectively). Values of initial preoperative neutral position and after anterior scalenotomy regarding the deltoid muscle, the biceps brachii muscle, the triceps brachii muscle and the abductor digiti minimi muscle 2 were similar (*p* > 0.05), while for the abductor digiti minimi muscle 1, its values after anterior scalenotomy tended to be lower by 63% compared to the initial preoperative neutral position (exponentiated β = 0.37, *p* = 0.056).

After taking into consideration the multiple comparisons and applying Holm-Bonferroni correction, it was found that the significant differences regarding deltoid, biceps brachii and triceps brachii muscles remained significant, while in abductor digiti minimi muscles 1 and 2 there were significant differences between final neutral position and after anterior scalenotomy.

Participants with prior surgery and those who had Double Crush Phenomenon were excluded, in terms of sensitivity analysis, and analyses were rerun in the remaining 23 patients. The results for deltoid, biceps brachii, triceps brachii and abductor digiti minimi 2 muscles were found similar. On the contrary, regarding the abductor digiti minimi muscle 1 it was found that the difference between final and initial neutral position was no longer significant [β (95% CI) = 0.185 (−0.047–0.416); exp β (95% CI) = 1.53 (0.90–2.61); *p* = 0.118].

## 4. Discussion

The term T.O.S is used to describe a constellation of symptoms resulting from the compression of the brachial plexus and the subclavian vessels at the thoracic outlet. It has been recognized in various forms for over 100 years. Understanding of the condition began with the identification of the cervical rib and its associated vascular complications, and progressed with the recognition of similar vascular symptoms in the absence of a cervical rib [10]. Cervical ribs tend to occur in approximately 0.5 to 1% of the population. The classification of cervical ribs proposed by Gruber (1869) remains particularly relevant in current clinical practice [11]. Four distinct types have been described. Type I cervical ribs extend only to the transverse process of the seventh cervical vertebra. In Type II, the cervical rib extends beyond the transverse process without any connection to the first rib. Type III cervical ribs extend beyond the transverse process and demonstrate partial fusion with the first rib through fibrous bands or cartilaginous connections. Type IV cervical ribs are completely fused with the first rib [11]. The underlying nature of T.O.S, the absence of reliable and valid diagnostic tests, and the high potential for major surgical complications initially rendered its surgical treatment controversial. Currently, however, surgery is considered as the definitive treatment for the syndrome, while conservative approaches—namely physical therapy in combination with analgesic medication—and their duration are increasingly questioned [12].

T.O.S is classified into neurogenic (90–95%), vascular (arterial and/or venous), and mixed (a combination of the two) types [13]. Diagnosis is challenging and requires a high index of clinical suspicion. It is based on the patient’s history, physical examination, and a comprehensive laboratory workup, including radiographic evaluation (chest and cervical spine X-rays, cervical spine and brachial plexus MRI, color Doppler ultrasonography (triplex) of the subclavian vessels, and when indicated, arteriography and/or venography), as well as specialized electrophysiological testing of brachial plexus function. Misdiagnosis may lead to the patient undergoing a demanding surgical procedure without the desired outcomes. Surgical interventions aim at decompressing and functionally restoring the affected structures and may involve scalenotomy, partial or complete first rib resection, and removal of any cervical rib if present.

Surgical approaches include transaxillary, supraclavicular, infraclavicular, posterior thoracoplastic, combined dual approaches (e.g., transaxillary with posterior incision), and thoracoscopic-robotic techniques, each associated with distinct complication profiles. According to systematic reviews, the transaxillary approach with concomitant first rib resection is associated with pneumothorax in approximately 14% of patients, neurological injury in 5%, vascular injury in 0.4%, and an overall mortality rate of 0.04% [13]. The supraclavicular approach with first rib resection has been reported to carry a pneumothorax rate of 19%, neurological complications in 3%, vascular injury in 0.3%, and mortality in 0.07% of cases. In contrast, supraclavicular decompression without first rib resection has been associated with pneumothorax in 5%, neurological injury in 7%, vascular injury in 0.2%, and no mortality [14]. The infraclavicular approach has been reported to result in pneumothorax in 7% of patients, with no neurological injuries observed [15]. Comparable complication rates have been described in combined techniques, such as thoracoscopically assisted transaxillary decompression, where pneumothorax occurred in 3% and neurological injury in 2% [16]. Purely thoracoscopic approaches were associated with pleural entry in all cases (100%), while neurological complications were reported in 3% [17]. Finally, robotic first rib resection has demonstrated neurological complication rates of up to 3.4% [18] and vascular injury in fewer than 5% of cases [19], whereas conversion to open surgery has been reported in 0–2% of patients [19]. Among these surgical approaches, the transaxillary approach is considered superior, as it allows for both scalenotomy and complete first rib resection, avoids pleural cavity entry, provides satisfactory cosmetic results, and—particularly when performed by an experienced surgical team-minimizes the risk of serious complications due to injury of the subclavian vessels or the brachial plexus [20,21,22].

With regard to novel approaches in the management of T.O.S, the thoracoscopic technique provides excellent visualization of the first rib, particularly its posterior segment, while avoiding the need for division of major muscles during access as well as retraction of the anatomical structures in the region [23]. This allows for an extensive rib resection with low rates of vascular and neurological complications [17]. Moreover, thoracoscopic surgery is associated with reduced postoperative pain, favorable cosmetic outcomes, and shorter hospital stays [17]. An additional advantage lies in its value as a teaching tool, offering an effective platform for the training of young surgeons [17].

The robotic approach further enhances the advantages of thoracoscopic surgery by providing three-dimensional visualization and improved ergonomics, including greater movement stability for the surgeon within a predominantly confined anatomical region [24]. Studies report minimal intraoperative blood loss, a very low rate of conversion to open approaches, reduced postoperative pain, and a low overall complication rate [25]. However, the robotic approach is limited in its clinical applicability and is associated with high costs.

The transaxillary approach for first rib resection, performed in conjunction with anterior scalenotomy, has demonstrated consistent efficacy and safety in the surgical management of T.O.S [8]. A report of a 30-year experience involving 103 patients treated with the rib resection technique demonstrated a low symptom recurrence rate of 5.8%, with a mean follow-up duration of 93 months [26]. Another systematic review reported a success rate exceeding 70% [14]. Similarly even higher rates were observed in a single-center study of 56 patients, which were part of a larger 19-year experience that included 1154 procedures in 818 patients. In that cohort, symptom resolution was achieved in 92.8% of patients with a mean follow-up period of 591 days, with only 7.1% of patients requiring further evaluation for residual symptoms or pain management [27]. Additionally, a separate study of 82 patients who underwent transaxillary first rib resection and anterior scalenotomy reported symptom resolution in 80.5% of cases, with residual symptoms in just 17.1% of patients [28]. Another large-scale study further confirmed the effectiveness of the transaxillary approach, reporting clinical improvement or complete symptom resolution in 90% of 308 studied patients [29]. Collectively, these findings support the transaxillary approach as a reliable and effective surgical option for T.O.S, with favorable long-term outcomes and significant functional recovery.

The transaxillary first rib resection, performed following anterior scalenotomy and division of the costoclavicular ligament, is the surgical approach adopted by our department for the treatment of all types of T.O.S. This approach is supported by evidence demonstrating that first rib resection is superior to anterior scalenotomy as a definitive treatment for the condition [30]. The diagnosis of T.O.S was based on a detailed patient history and clinical examination, including Adson’s test, Wright’s test, Roos’ test, and palpatory provocative maneuvers at scalene triangle and pectoralis minor insertion. In addition, all patients underwent imaging studies, including chest and cervical spine X-rays, cervical spine MRI, and upper limb Duplex ultrasonography. The increased width of the first rib, along with its vertical orientation as assessed on the preoperative chest radiograph, was associated intraoperatively with a greater degree of compression of both the subclavian artery and the brachial plexus. The diagnostic workup was completed with upper limb electromyography. All available diagnostic assessments support the clinical suspicion of the syndrome. Of particular interest is the presence of a clinical phenotype consistent with neurogenic T.O.S, which, however, is not corroborated by electromyographic findings. In the context of longitudinal patient management—especially for those reluctant to undergo surgical intervention—brachial plexus surveillance is proposed as a key component of ongoing monitoring. Further indications for neuro-monitoring are well-documented in the literature, including cases of trauma involving the clavicle or first rib, where clinical evaluation continues to serve as the primary diagnostic tool [31,32]. During the procedure, elevation of the upper limb is of paramount importance in order to protect the brachial plexus and the subclavian artery during surgical maneuvers. Anterior scalenotomy is performed by dividing the muscle close to its insertion on the first rib to minimize the risk of phrenic nerve injury, given that the nerve runs anteriorly along the surface of the anterior scalene before entering the thoracic cavity. A transient phrenic nerve injury (elevated hemidiaphragm) occurred in one patient, likely due to thermal damage from monopolar electrocautery, which resolved completely within six days. To avoid injury to the long thoracic nerve and subsequent development of scapular winging, the middle scalene muscle is carefully retracted posteriorly. In patients with a supernumerary cervical rib, the bony variation was usually ipsilateral to the symptomatic upper limb. Resection was performed as posteriorly as possible, near its articulation with the transverse process, to achieve complete removal while minimizing neurovascular risk. To date, no recurrences have been observed, as both first rib and cervical rib remnants are small. Decompression of the subclavian vein in venous T.O.S is achieved by dividing the costoclavicular ligament, performed adjunctively with anterior scalenotomy and first rib resection, as the vein lies external to the scalene triangle and is particularly vulnerable to extrinsic compression at this site. This procedure does not require specialized equipment. Deep retractors, a long osteotome, a rongeur (to refine the cut edges of the rib) and standard monopolar cautery are sufficient to complete the operation. A helpful adjunct is the use of a single-use LED light source attached to the retractor, which significantly improves visibility in the narrow and deep surgical field. This approach is based on the department’s extensive experience in managing the condition and highlights the necessity of maximal decompression of the thoracic outlet structures from any anatomically driven source of compression. Moreover, due to the close anatomical relationship between the brachial plexus and the subclavian artery at the thoracic outlet, neurologic and arterial forms of T.O.S often present with overlapping or mixed-type clinical features. Consequently, our surgical approach does not differ according to the T.O.S subtype. This uniform strategy contributes to the reduction in disease recurrence and the need for reoperation. Reoperations are considered particularly demanding due to the formation of adhesions and fibrous bands in this anatomically complex region. Additionally, the aforementioned technique has been proven effective in treating recurrent cases of T.O.S that were previously managed elsewhere via a supraclavicular anterior scalenotomy without a first rib resection.

The principal strength of the present study lies in the fact that, to the best of our knowledge, no comparable intraoperative documentation of brachial plexus decompression has previously been reported in the international literature. Conversely, certain limitations must be acknowledged, including the single-center nature of the patient cohort and the absence of a double-blind study design. The latter was a deliberate choice, as we strongly advocate for the superiority of transaxillary first rib resection following anterior scalenotomy, which we consider the optimal surgical option for our patients. This perspective is consistent with the primary aim of the study, namely, to evaluate the effect of first rib resection in achieving complete decompression of the brachial plexus in the operative management of T.O.S. Τhe present analysis relies on changes in MEP amplitude as an indirect surrogate of neural conduction across the brachial plexus, rather than as a direct measure of brachial plexus decompression. Our results are consistent with this approach, as demonstrated through intraoperative electromyographic recordings. Overall, muscle activity was markedly increased at the final postoperative neutral position across multiple upper limb muscles. Specifically, deltoid, biceps brachii, and triceps brachii values were significantly higher at the final postoperative position compared both to measurements obtained after anterior scalenotomy and to the initial preoperative neutral position, highlighting the effectiveness of full surgical decompression in restoring proximal muscle function. Similarly, the abductor digiti minimi muscles 1 and 2 exhibited substantial increases at the final postoperative position compared to post-scalenotomy and preoperative measurements. Notably, abductor digiti minimi 1 showed a trend toward decreased activity after anterior scalenotomy alone, although this difference was no longer significant after full decompression, as confirmed in the sensitivity analysis excluding patients with prior surgery or Double Crush Phenomenon. This observation may represent either the necessity of first rib resection to ensure adequate decompression of the lower brachial plexus roots or transient nerve irritation occurring during intraoperative manipulations. Such manipulations include the anterior scalenotomy itself, as well as posterior retraction of the middle scalene muscle, performed to prevent injury to the long thoracic nerve. In contrast, abductor digiti minimi 2 values remained stable between preoperative and post-scalenotomy measurements. These findings indicate that anterior scalenotomy alone may transiently impair distal nerve function in specific muscles, whereas complete decompression, including first rib resection, effectively restores the muscle activity. Finally, correlating intraoperative assessment of brachial plexus decompression with postoperative clinical outcomes would provide additional insight. This analysis is currently ongoing for the patients included in the present study.

## 5. Conclusions

Intraoperative electromyographic monitoring demonstrates a significant improvement in brachial plexus function following first rib resection during the Roos procedure, whereas minimal changes or possible transient deterioration are observed in the monitored muscles after isolated anterior scalenotomy in the surgical position. These findings provide physiological evidence supporting the practice of comprehensive thoracic outlet decompression, including first rib resection. Nevertheless, comparative studies with alternative surgical approaches are required to definitively establish relative efficacy. This monitoring technique may also be valuable for the intraoperative assessment of decompression adequacy.

## Figures and Tables

**Table 1:** Patients’ Characteristics.

*n* = 34		*n*	%
Sex	Males	6	17.6
	Females	28	82.4
Thoracic Outlet Syndrome Type	Neurogenic	10	29.4
	Arterial	7	20.6
	Mixed	15	44.1
	Venous	2	5.9
Years with symptoms	<5	22	64.7
	5–10	4	11.8
	>10	8	23.5
Physiotherapy pre-operatively	No	18	52.9
	Yes	16	47.1
Sports activities	No	20	58.8
	Yes	14	41.2
Limb	Right	19	55.9
	Left	15	44.1
Multiple pressure syndrome	No	28	82.4
	Yes	6	17.6
Reoperation transaxillary resection of the first rib	No	34	100.0
	Yes	0	0.0
Scalenotomy reoperation	No	28	82.4
	Yes	6	17.6
Cervical spine pathology	No	14	41.2
	Yes	20	58.8
Upper extremity-cervical spine injuries	No	27	79.4
	Yes	7	20.6
		Mean	SD
Age (years)		34.6	10.7

**Table 2:** Clinical presentation.

*n* = 34	*n*	%
Pain related symptoms		
Shoulder girdle pain	19	55.9
Neck pain	11	32.4
Chest wall pain	11	32.4
Arm pain	21	61.8
Forearm pain	21	61.8
Hand pain	17	50.0
Neurological symptoms		
Paresthesia–numbness	30	88.2
Upper limb weakness	10	29.4
Muscle atrophy	1	2.9
Headache	6	17.6
Vascular symptoms		
Change in limb temperature	11	32.4
Change in limb color	15	44.1
Upper limb edema	12	35.3
Prominent superficial veins	2	5.9

**Table 3:** Patients’ muscle measurements.

		*n*	Mean (SD)	Median (IQR)
Deltoid muscle (μV)	Initial neutral position	34	56.54 (78.64)	27.45 (8.6–74.6)
	After anterior scalenotomy	26	57.56 (86.6)	29.65 (1.4–99.2)
	Final neutral position	33	176.15 (208.98)	80.7 (38.3–277)
	Change from initial to final neutral position	33	116.21 (211.76)	48.1 (2.1–164.3)
Biceps brachii muscle (μV)	Initial neutral position	34	100.32 (152.24)	51.55 (2.5–123)
	After anterior scalenotomy	26	70.83 (89.54)	25.7 (5.1–130)
	Final neutral position	33	291.69 (367.62)	155 (59.5–376)
	Change from initial to final neutral position	33	189.39 (316.7)	68.7 (0.78–284.4)
Triceps brachii muscle (μV)	Initial neutral position	34	72.38 (105.11)	37.9 (1.7–123)
	After anterior scalenotomy	26	48.9 (53.75)	37.7 (1.9–86.1)
	Final neutral position	33	166.95 (217.64)	98.8 (32.8–184)
	Change from initial to final neutral position	33	93.43 (216.13)	35 (6.9–133.5)
Abductor digiti minimi muscle 1 (μV)	Initial neutral position	34	321.98 (334.08)	187.5 (98.7–454)
	After anterior scalenotomy	26	240.04 (338.81)	108.5 (36.1–286)
	Final neutral position	33	458.23 (437.08)	211 (96–810)
	Change from initial to final neutral position	33	138.55 (318.6)	32 (−7.6–245)
Abductor digiti minimi muscle 2 (μV)	Initial neutral position	34	179.22 (353.22)	8.85 (1.7–184)
	After anterior scalenotomy	26	183.11 (333.04)	5.45 (3.1–268)
	Final neutral position	33	225.34 (379.7)	45.5 (2.5–232)
	Change from initial to final neutral position	33	40.95 (214.81)	4.86 (−1.06–55.3)

**Table 4:** Mixed linear models results with muscle measurements as dependent variables.

Muscle	Measurements	β (95% CI)+	Exponentiated Coefficient (95% CI)	*p*
Deltoid muscle (μV)	After anterior scalenotomy vs. Initial neutral position	0.041 (−0.250–0.332)	1.10 (0.56–2.15)	0.784
	Final neutral position vs. Initial neutral position	0.608 (0.341–0.875)	4.06 (2.19–7.50)	*<0.001*
	Final neutral position vs. After anterior scalenotomy	0.570 (0.319–0.821)	3.72 (2.08–6.62)	*<0.001*
Biceps brachii muscle (μV)	After anterior scalenotomy vs. Initial neutral position	0.090 (−0.267–0.447)	1.23 (0.54–2.80)	0.622
	Final neutral position vs. Initial neutral position	0.541 (0.208–0.873)	3.48 (1.61–7.46)	*0.001*
	Final neutral position vs. After anterior scalenotomy	0.446 (0.255–0.636)	2.79 (1.8–4.33)	*<0.001*
Triceps brachii muscle (μV)	After anterior scalenotomy vs. Initial neutral position	−0.182 (−0.540–0.177)	0.66 (0.29–1.50)	0.321
	Final neutral position vs. Initial neutral position	0.455 (0.230–0.680)	2.85 (1.70–4.79)	<0.001
	Final neutral position vs. After anterior scalenotomy	0.606 (0.302–0.910)	4.04 (2.00–8.13)	<0.001
Abductor digiti minimi muscle 1 (μV)	After anterior scalenotomy vs. Initial neutral position	−0.431 (−0.873–0.012)	0.37 (0.13–1.03)	0.056
	Final neutral position vs. Initial neutral position	0.214 (0.002–0.426)	1.64 (1.00–2.67)	0.048
	Final neutral position vs. After anterior scalenotomy	0.672 (0.307–1.039)	4.7 (2.03–10.94)	*<0.001*
Abductor digiti minimi muscle 2 (μV)	After anterior scalenotomy vs. Initial neutral position	−0.056 (−0.255–0.144)	0.88 (0.56–1.39)	0.584
	Final neutral position vs. Initial neutral position	0.268 (0.043–0.494)	1.85 (1.10–3.12)	0.020
	Final neutral position vs. After anterior scalenotomy	0.317 (0.088–0.547)	2.07 (1.22–3.52)	0.007

Note: The logarithmic transformations were used for this analysis; *p*-values in italics denote significant findings after applying Holm-Bonferroni correction for multiple comparisons +regression coefficient (95% Confidence Interval).

## Data Availability

Data access is strictly limited to the authors specifically as regulated by authority approvals, and therefore not possible to share.

## Abbreviations

T.O.S	Thoracic Outlet Syndrome.
SSEPs	Somatosensory Evoked Potentials.
MEPs	Motor evoked potentials.
MRI	Magnetic Resonance Imaging.
EMG	Electromyography.
PMS	Pectoralis Minor Syndrome.
DCP	Double Crush Phenomenon.
Pre-op	Preoperative.
Post-Scalenotomy	After anterior Scalenotomy
